# The MTA score—simple and reliable, the best for now?

**DOI:** 10.1007/s00330-021-08340-8

**Published:** 2021-10-02

**Authors:** Alexander Rau, Horst Urbach

**Affiliations:** Department of Neuroradiology, Medical Center – University of Freiburg, Faculty of Medicine, University of Freiburg, Breisacher Str. 64, 79106 Freiburg, Germany

Dementia is becoming increasingly important against the background of demographic change with Alzheimer’s disease (AD) as the most common cause [1]. Early and valid diagnosis is desirable, especially in view of emerging causal therapies at the horizon; e.g., the amyloid beta–directed antibody aducanumab has been approved by the FDA in June 2021.

Current efforts of the National Institute on Aging—Alzheimer’s Association aim at finding biomarkers in order to identify patients at risk to develop AD [1]. While cerebrospinal fluid and PET biomarkers detect the amyloid and tau burden, MRI is considered to capture the pathological cascade at a later point of time when neuronal damage and atrophy have already occurred. In daily practice, however, MRI is mainly used to exclude potentially treatable causes of dementia (i.e., subdural hematoma, normal pressure hydrocephalus, tumor) [2].

MRI and—with a lesser accuracy—CT have a particular value as widely available and noninvasive examination techniques. Typical atrophy patterns correlate with various pathologies and in prodromal stages such as minimal cognitive impairment, mesiotemporal atrophy (MTA) correlates with conversion to AD [3]. The MTA score, published by Scheltens and colleagues in 1992 [4], is a simple measure by which mesiotemporal atrophy can be quantified. Using the width of the choroidal fissure, temporal horn, and height of the hippocampal formation, atrophy is evaluated in five grades (0–4). Score 0 indicates no atrophy, score 1 indicates widening of the choroidal fissure, score 2 includes additional widening of the temporal horn of the lateral ventricle and slightly decreased hippocampal formation height, score 3 includes moderate loss of hippocampal formation volume, and 4 indicates an increase in all of these findings in the final stage.

Interestingly, in recent publications, only the hippocampal height itself is often assessed, whereas in the initial publication, the entire hippocampal formation (defined as dentate gyrus, hippocampus proper, and subiculum together with parahippocampal gyrus) was rated.

Other important scores for the evaluation of AD-typical atrophy patterns are the parietal atrophy score [5] and the ERICA score which focusses on the entorhinal cortex and not the hippocampus [6]. The rationale behind this is the fact that AD-typical tau depositions start in the entorhinal cortex and not the hippocampus. Even if these scores are not directly assessed, the image impression is at least unconsciously included in the evaluation.

MTA itself is typical of AD but not specific and is also observed in frontotemporal dementia, for example. Parietal atrophy provides important additional information in the assessment of MTA, as MTA in combination with parietal atrophy is more suggestive of AD than MTA alone [5].

In a meta-analysis, Park and colleagues demonstrated a pooled sensitivity of 74% and 84% specificity for the MTA score in differentiating AD patients from healthy controls [7]. In addition, a high inter- and intraobserver correlation was observed. The diagnostic value for the differentiation of AD from other neurodegenerative diseases was found to be fundamentally lower.

A general problem with the MTA score is the inconsistently defined cutoff value. Various cutoffs for pathological MTA scores can be found in the literature, differing by age groups and education level. For example, Velickaite and colleagues [8] elaborated that “at age 75, gender and education are confounders for MTA grading. A score of ≥ 2 is abnormal for low-educated women and a score of ≥ 2.5 is abnormal for men and high-educated women.” For this, the mean for both sides was considered together ((MTA score right + MTA score left)/2).

In the meta-analysis, the included studies differed in the cutoff values used. Furthermore, the method of MTA determination (worse side vs. mean) had an effect on heterogeneity among the studies as proven by the meta-regression. Nevertheless, satisfactory performance of the MTA score was confirmed.

An inferiority of the MTA score compared to hippocampal volumetry could not be shown. The advantages of the MTA score, which is readily available and can be determined free of charge, are obvious. However, clinically usable postprocessing algorithms (with the results available when the MRI scan is finished) will likely supersede semiquantitative scores like the MTA score in the near future. They objectify the visual impression of the examiner by voxel- and region-based comparisons of the individual MRI scan with huge data bases of age-matched healthy controls [9]. Artificial intelligence–based techniques can better assign atrophy patterns to different pathologies. While first results are promising [10], further evaluation and establishment is still pending. For the use outside of specialized centers, free tools are available (e.g., https://github.com/BrainImAccs/veganbagel).

The authors also raised the interesting discussion point that the level of coronal slice used for MTA scoring has not been conclusively defined! However, the image impression depends on the selected slice and a standardized assessment is desirable for the wide application. In the ERICA score, for example, the level of the mamillary bodies is given as a reference.

Probably limited by the available number of studies, a difference in performance of the MTA score in MRI compared to CT could not be demonstrated. Though, the detection of atrophy is recommended in a 3D T1w sequence [6].

Even though structural imaging is still primarily used as a diagnostic tool to exclude treatable causes of dementia, there is an additional diagnostic value in identifying the underlying cause of dementia.

With MRI and CT, noninvasive biomarkers for neurodegenerative diseases are widely available. This work contributes to the establishment of the easy-to-measure MTA score in the broad field of radiology and not only in neuroradiology and specialized centers.

Since structural imaging is recommended anyway, let’s get it right and evaluate the cause of dementia beyond treatable conditions. The MTA score is a valuable and reliable tool but maybe not for the entire future.

## Declarations

### Guarantor

The scientific guarantor of this publication is Prof. Horst Urbach.

### Statistics and biometry

No complex statistical methods were necessary for this paper.

### Methodology

• Editorial comment.
